# Efficacy and safety of Omadacycline in community-acquired pneumonia among elderly patients: a real-world evidence study

**DOI:** 10.3389/fcimb.2026.1875598

**Published:** 2026-07-31

**Authors:** Xinran Xie, Jiayu Deng, Gengyuan Sun, Yanqing Song

**Affiliations:** 1 School of Pharmaceutical Sciences, Jilin University, Changchun, China; 2 Department of Pharmacy of Lequn Branch, The First Hospital of Jilin University, Changchun, China

**Keywords:** community-acquired pneumonia, elderly patients, immunocompromised individuals, inverse probability of treatment weighting, omadacycline, real-world study

## Abstract

**Objective:**

This study aimed to evaluate real-world effectiveness and safety of omadacycline for elderly community-acquired pneumonia (CAP) versus tigecycline, moxifloxacin, and cefoperazone-sulbactam after inverse probability of treatment weighting (IPTW).

**Methods:**

This single-center retrospective cohort study enrolled non-ICU hospitalized elderly CAP patients. Missing covariates were handled by multiple imputation, and IPTW balanced groups. Primary outcome was pragmatic clinical response (improvement in at least one of laboratory markers, chest imaging, or clinical symptoms), with a sensitivity analysis requiring all three. Secondary outcomes included hospital stay, antibiotic duration, microbiological clearance, and adverse events. Subgroup analyses used age < 75 vs ≥ 75 years and CURB-65 < 2 vs ≥ 2.

**Results:**

A total of 1,208 patients were included: 177 in the omadacycline group, 124 in the tigecycline group, 666 in the moxifloxacin group, and 241 in the cefoperazone-sulbactam group. After IPTW adjustment, omadacycline showed a pragmatic clinical response comparable to tigecycline, higher than moxifloxacin, but lower than cefoperazone-sulbactam. Omadacycline was associated with shorter hospital stay than tigecycline and cefoperazone-sulbactam, but longer than moxifloxacin; antibiotic duration was comparable to tigecycline, shorter than cefoperazone-sulbactam, but longer than moxifloxacin. Microbiological clearance was lower with omadacycline in the evaluable subset, whereas recorded adverse events were fewer but limited by rare-event sparsity. Exploratory subgroup analyses suggested that cefoperazone-sulbactam had a higher response than omadacycline in patients aged <75 years or with CURB-65 <2. Subgroup analyses suggested heterogeneous associations by age and disease severity and should be interpreted as exploratory.

**Conclusion:**

In this real-world analysis of elderly patients with CAP after IPTW adjustment, omadacycline showed a pragmatic clinical response comparable to tigecycline, higher than moxifloxacin, but lower than cefoperazone-sulbactam. Omadacycline was associated with shorter hospital stay than tigecycline and cefoperazone-sulbactam, but longer hospital stay than moxifloxacin. Antibiotic duration was comparable to tigecycline, shorter than cefoperazone-sulbactam, but longer than moxifloxacin. Given the broad pragmatic endpoint, high baseline response rate, sensitivity to endpoint definition and missing-data handling, incomplete microbiological follow-up, and residual confounding inherent to retrospective data, these findings should be interpreted as exploratory and require prospective validation.

## 1 Introduction

Community-acquired pneumonia (CAP) is one of the most common infectious diseases, with millions of elderly patients worldwide admitted to hospitals each year. Its incidence and mortality rates increase significantly with advancing age (Dequin et al., 2026). Older adults constitute a classic immunocompromised cohort. This is due to immunosenescence, multiple coexisting conditions, and diminished physiological reserve capacity. These factors significantly elevate the risk of developing community-acquired pneumonia (Cillóniz et al., 2020). According to epidemiological data, community-acquired pneumonia is one of the primary causes of hospitalization and mortality among the elderly (Li et al., 2025). The clinical presentation in such patients is often atypical, with more severe disease, increased complications, prolonged hospital stays, and a significantly higher risk of mortality than in younger patients (Elias et al., 2024). Therefore, community-acquired pneumonia (CAP) in the elderly not only imposes a significant burden on global public health systems and individual well-being, but also places heightened demands on clinical anti-infective treatment (Shi et al., 2020).

Currently, the management of community-acquired pneumonia in the elderly presents multiple challenges. First, elderly patients frequently exhibit reduced hepatic and renal function, which directly impacts the metabolism and excretion of antimicrobial agents. This not only increases the risk of drug accumulation and toxicity but also restricts the safe use of certain medications (Soraci et al., 2023). Second, the elderly population inherently exhibits compromised immune function due to immunosenescence, often alongside multiple underlying conditions. The inappropriate use of broad-spectrum antibiotics exacerbates the emergence of multidrug-resistant pathogens, posing a significant global challenge (Mancinetti et al., 2024). Therefore, there is an urgent clinical need for a novel antimicrobial agent that combines broad-spectrum efficacy with good safety, high oral bioavailability, and minimal impact on hepatic and renal functions. This would meet the requirements for individualized treatment in older adults with community-acquired pneumonia and optimize anti-infective strategies (Ferreira-Coimbra et al., 2020; Putot et al., 2025).

At present, empirical treatment for CAP primarily relies on broad-spectrum antimicrobial agents (Metlay et al., 2019). Omadacycline is a novel oxamethyltetracycline antibacterial agent whose structural modification enables it to overcome resistance mediated by common tetracycline efflux pumps and ribosomal protection. It exhibits *in vitro* antibacterial activity against common CAP pathogens, such as Streptococcus pneumoniae, Haemophilus influenzae, Moraxella catarrhalis, and atypical pathogens (Stets et al., 2019). Its pharmacokinetic properties are favorable, characterized by high oral bioavailability, strong tissue penetration, and primary elimination via feces. It exhibits minimal renal dependency and does not require dosage adjustment in patients with hepatic impairment (Rodvold and Pai, 2019). These characteristics are theoretically well-suited for elderly patients with CAP requiring outpatient oral therapy or those with potentially impaired hepatic or renal function. Although studies have demonstrated that omadacycline is more effective than moxifloxacin in adult and elderly patients with CAP, with generally favorable overall safety, comparative studies against other antibiotics have predominantly focused on younger adult and adolescent patient populations (Yu et al., 2025). The physiological characteristics of the elderly population, along with the prevalence of multiple coexisting conditions and polypharmacy, may influence the actual efficacy of medications. These factors cannot be fully extrapolated from registration clinical trials that predominantly involve middle-aged and younger adults.

Therefore, this study employed a retrospective cohort design and utilized IPTW to compare the efficacy and safety of omadacycline versus tigecycline, moxifloxacin, and cefoperazone-sulbactam in elderly patients with community-acquired pneumonia. The aim was to provide optimized empirical antimicrobial treatment options for immunocompromised elderly populations and furnish evidence-based guidance for individualized clinical prescribing decisions.

## 2 Methods

### 2.1 Study design and data source

This was a single-center, retrospective, real-world comparative-effectiveness cohort study in non-ICU hospitalized elderly patients with community-acquired pneumonia (CAP) at the Lequn Branch, The First Hospital of Jilin University, a tertiary academic medical center. The study period spanned from 1 August 2024 to 31 December 2025.

Patient-level data were obtained from the hospital’s health information system (HIS). Extracted variables included demographic information (age, gender), clinical diagnoses, medication orders (drug name, dosage, frequency, duration), and laboratory test results such as white blood cell count (WBC), neutrophil percentage (NE%), procalcitonin (PCT), serum creatinine (SCr), C-reactive protein (CRP), interleukin-6 (IL-6), aspartate aminotransferase (AST), alanine aminotransferase (ALT), microbiological test reports, NGS reports, and chest CT findings. To ensure consistency in data collection and endpoint classification, all relevant data were extracted into a structured case report form (CRF).

All data were fully anonymized before analysis. This study was approved by the Ethics Committee of The First Hospital of Jilin University (Approval No.2026-085). As a retrospective design using fully anonymized administrative and clinical data, the requirement for informed consent was waived.

### 2.2 Patient selection and grouping assignment

This study included hospitalized patients aged ≥ 65 years who were diagnosed with CAP. To ensure adequate therapeutic exposure, patients were administered omadacycline, tigecycline, moxifloxacin, or cefoperazone-sulbactam for at least 72 h. Exclusion criteria were established to reduce heterogeneity and ensure data completeness: (i) initial antibiotic treatment duration < 72 hours, (ii) non-bacterial pneumonia (e.g., viral pneumonia), and (iii) concomitant pulmonary or infectious diseases. All data were ascertained through comprehensive electronic medical record review, including diagnostic codes, laboratory values, imaging findings, and medication details.

**Figure 1:**
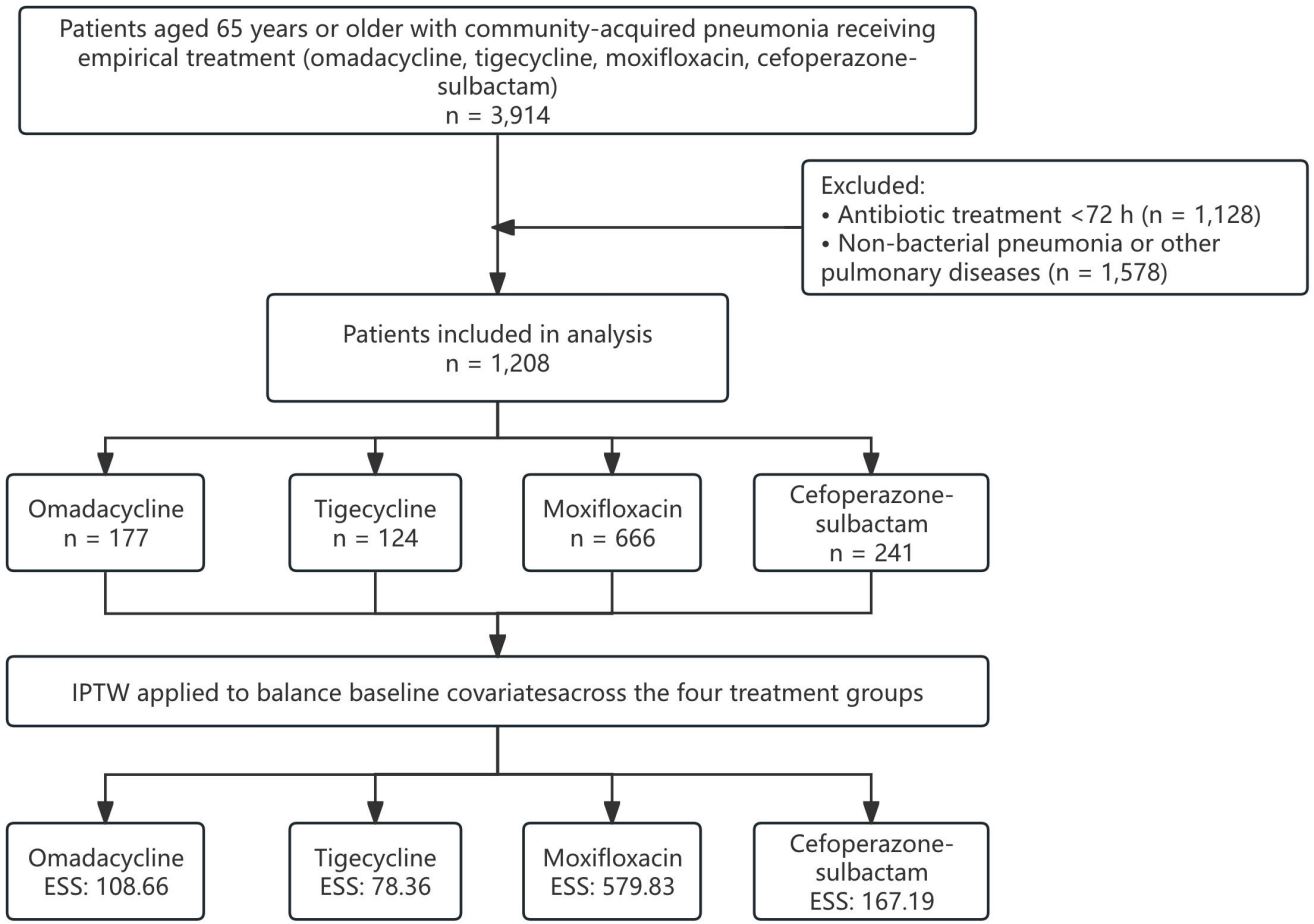
Flow diagram of patient screening, multinomial IPTW cohort selection, and stabilized effective sample size (ESS) construction. Description: This flowchart systematically illustrates the structured patient selection, clinical exclusion pathways, and the final pseudo-population derivation for non-ICU hospitalized elderly individuals diagnosed with community-acquired pneumonia (CAP). From an initial pool of 3,914 patients, a final pre-weighting comparative cohort of 1,208 individuals was established after removing patients with brief therapeutic exposures (<72 hours) or non-bacterial clinical presentations. Following the allocation of stabilized multinomial inverse probability of treatment weighting (IPTW) to achieve baseline covariate balance, the design-effect-adjusted Effective Sample Size (ESS) for each active anti-infective arm is explicitly detailed (Omadacycline ESS = 108.66; Tigecycline ESS = 78.36; Moxifloxacin ESS = 579.83; Cefoperazone-sulbactam ESS = 167.19). The stabilized headcounts represent the true, uninflated statistical power utilized for subsequent multi-group inferential modeling.

Eligible patients were assigned to one of four treatment groups based on the empirical antibiotic regimen they had started: omadacycline, tigecycline, moxifloxacin, or cefoperazone-sulbactam. Patients who changed antimicrobial therapy within the preceding 72 h were excluded to ensure that the treatment allocation reflected the initial evidence-based clinical decision. Patients receiving omadacycline constituted the intervention group, whereas those receiving any of the other three antibiotics formed the control group. To minimize indication bias and enhance comparability across the four treatment groups, an IPTW method based on baseline characteristics was employed. The process of patient identification, exclusion and construction of the final cohort is shown in **Figure 1**.

### 2.3 Definition of outcomes

Primary outcome (pragmatic clinical response): The primary outcome was a pragmatic clinical response during hospitalization, defined as documented improvement in at least one of the following domains by discharge: (i) laboratory infection markers (e.g., WBC, CRP, PCT) showing normalization or clear downward trend compared with baseline; (ii) chest CT/radiology report indicating absorption/resolution of inflammatory lesions or reduced extent; or (iii) clinical symptoms (fever, cough, sputum) documented as improved/resolved in progress notes. To avoid misclassification, outcome assessment was performed through a structured chart review by two investigators with adjudication of disagreements. Because this endpoint was defined among non-fatal hospitalizations, in-hospital mortality was analyzed as a separate outcome.

Secondary outcomes included length of hospital stay, duration of antibiotics, microbiological clearance, and adverse events. Microbiological clearance was assessed only among patients with available follow-up cultures after the initiation of empirical therapy because repeat cultures were not routinely obtained for all patients. Clearance was defined as a negative follow-up culture, with no growth of the baseline pathogen, documented before discharge. Because follow-up cultures were ordered according to routine clinical judgment rather than a standardized protocol, this endpoint was considered exploratory and restricted to a microbiologically evaluable subset. Accordingly, the denominators for microbiological analyses differed across treatment groups and are explicitly reported in the Results.

Safety endpoints focused on the incidence of drug-related adverse events (AEs) that occurred during the duration of active antibiotic therapy. To ensure rigorous ascertainment within this retrospective design, AEs were identified through a structured electronic chart extraction protocol scanning standardized safety terminologies, laboratory alerts (e.g., severe liver or renal enzyme elevations), and free-text clinician progress notes. All recorded historical events were clinically adjudicated based on the Common Terminology Criteria for Adverse Events (CTCAE, version 5.0).

### 2.4 Covariates and data collection

Baseline covariates were selected based on clinical relevance and potential impact on treatment choices or outcomes. These covariates included sex, age, height, weight, BMI, smoking status, COPD, and CURB-65 score. All covariates were measured prior to or at the commencement of empirical antibiotic therapy to ensure the correct temporal sequencing for propensity score estimation.

### 2.5 Propensity score estimation and inverse probability of treatment weighting

To reduce measured baseline imbalance and potential confounding in this observational comparison, we employed an IPTW approach based on propensity scores. Propensity scores were estimated using a multinomial logistic regression model, with the four treatment groups as the dependent variable and age, sex, height, weight, BMI, smoking history, coexisting COPD, and CURB-65 score as covariates. All covariates were measured before the initiation of empirical treatment to preserve the temporal sequence required for propensity score estimation.

For each patient, the model provided an estimated probability of receiving the actual treatment. Stabilized IPTW weights were subsequently constructed to estimate the ATE. The stabilized weight was defined as:


$$SW_{i}=\frac{P\left(T=T_{i}\right)}{{\hat{e}}_{T_{i}}\left(X_{i}\right)}$$

where *P*(*T* = *T_i_*) denotes the marginal probability of receiving treatment *t*, and ˆei(t) is the generalized propensity score for patient i. Stabilized weights help preserve the sample size and improve estimator efficiency while reducing the influence of extreme values, as recommended in the marginal structural model methodology.

### 2.6 Assessment of covariate balance

Covariate balance before and after weighting was evaluated using standardized mean differences (SMDs) instead of p-values. For continuous variables, the SMDs were calculated as the absolute difference between the weighted means of each group divided by the pooled weighted standard deviation. For categorical variables, the SMDs were calculated as the absolute difference between the weighted proportions of each group divided by the pooled standard deviation.

To comprehensively evaluate the overall balance across the four groups, the maximum SMDs were computed for all treatment comparisons, and the maximum absolute pairwise SMD for each covariate was used to summarize the balance. An absolute SMD < 0.2 was prespecified to indicate adequate balance. Changes in the SMDs for all covariates before and after weighting were presented in the form of a LOVE plot. Concurrently, effective sample sizes (ESS) were calculated to assess weighting stability.

### 2.7 Missing data handling

Owing to missing data for certain covariates such as height, weight and BMI, multiple imputation methods were employed to address missing values, thereby minimizing bias and fully utilizing all available information. Multiple imputations were performed to address missing baseline covariates (height, weight, and BMI), generating five imputed datasets using a standard multiple imputation procedure implemented in R (MICE). The imputation model included baseline covariates, treatment groups, and outcomes. The estimates were pooled using Rubin’s rules. The imputation process preserved the intrinsic correlations between the variables while accounting for the uncertainty of the missing data. All subsequent analyses were conducted independently for each imputed dataset. The results from these datasets were pooled according to Rubin’s rules to derive the overall estimates and their standard errors.

### 2.8 Statistical analysis

Baseline liver function tests (alanine aminotransferase [ALT] and aspartate aminotransferase [AST]) were excluded from the multinomial propensity score framework because of profound missingness in real-world electronic triage (missing rates: 44.6% in the omadacycline group, 52.7% in the moxifloxacin group, 33.2% in the cefoperazone-sulbactam group, and 16.1% in the tigecycline group). To provide a quantitative sensitivity assessment for potential unmeasured confounding from omitted clinical covariates and other unavailable prognostic factors, such as baseline eGFR, phenotypic frailty, functional status, DNR/DNI orders, prior antibiotic exposure, aspiration risk, and microbiological severity, an E-value analysis was performed using the R ‘EValue’ package. Because E-value mathematical frameworks require ratio-based parameters, these values were calculated based on the adjusted odds ratios (OR) and their 95% confidence intervals derived from secondary weighted logistic regression models for the primary therapeutic comparisons.

Within each imputed dataset, baseline covariates were balanced across groups using IPTW, and the effect of omadacycline compared with the other three groups was estimated based on the weighted sample. For dichotomous outcomes such as the primary endpoint and microbial clearance rate, the weighted rates for each group, along with the risk difference (RD) between groups and its 95% confidence interval, were calculated. For continuous outcomes, such as length of hospital stay and duration of antibiotics, weighted means and between-group mean differences (MD) with 95% confidence intervals were calculated. All analyses employed robust standard errors to account for the weighting. The results from the five imputed datasets were combined using Rubin’s rule to derive pooled effect estimates, confidence intervals, and P-values. Prespecified subgroup analyses stratified by age (< 75/≥ 75 years) and CURB-65 score (< 2/≥ 2) were performed using the aforementioned IPTW analyses within each subgroup. Weighted regression models were used to test for interactions between the treatment group and subgroup variables (Wald test P<0.05, indicating significant interaction).

Furthermore, to stabilize the inverse probability of treatment weights (IPTW) against extreme rare-event data sparsity in the 30-day all-cause mortality analysis, a standard 1% and 99% weight trimming protocol was implemented to eliminate variance inflation from outlier weights. Weighted logistic regression models were fitted using generalized estimating equations (GEE) with a robust sandwich variance estimator. All statistical analyses were performed using R software (version 4.5.2). A two-tailed P value of < 0.05 was considered statistically significant, although clinical interpretation primarily relied on effect estimates and their confidence intervals.

To control the global Type I error rate inflated by executing multiple pairwise tests across various outcomes, a formal testing hierarchy was created. Pairwise comparisons for the primary efficacy endpoint were defined as confirmatory, whereas all secondary endpoints and stratified sub-models were explicitly classified as exploratory and hypothesis-generating. Crucially, a multiplicity correction via the Benjamini-Hochberg (BH) false discovery rate (FDR) procedure was applied to all 12 comprehensive pairwise comparisons. An adjusted q-value < 0.05 was designated as the threshold for maintaining statistical significance.The raw P-values and corresponding adjusted q-values are reported in **Table 1**.

**Table 1:** Primary and secondary outcome measures before and after adjustment for IPTW.

Outcome	Treatment	Unweighted (RD/MD, 95% CI)	Weighted (RD/MD, 95% CI)	P	q (FDR)	NNT (95% CI)
Cured/Improved (%) RD (95%CI)	Omadacycline (n=177)	163/174 (93.7)	93.2 (Reference)	N/A	N/A	N/A
	Tigecycline (n=124)	106/113 (93.8) RD, −0.13 (−5.9 to 5.6)	94.0 RD, −0.80 (−6.82 to 5.21)	0.782	0.782	N/A
	Moxifloxacin (n=666)	625/662 (94.4) RD, -0.73(−4.2 to 3.3)	94.5 RD, -1.3 (-6.4 to 3.8)	0.620	0.740	N/A
	Cefoperazone-sulbactam (n=241)	220/225 (97.8) RD, −4.1(−7.6 to 0.6)	98.1 RD, −4.16 (−8.29 to −0.03)	0.049	0.044	20 (16 to 62)
Length of hospital stay (days) MD (95%CI)	Omadacycline (n=177)	11.5 ± 7.1	12.1 (Reference)	N/A	N/A	N/A
	Tigecycline (n=124)	18.1 ± 10.6 MD, -6.59(-8.73 to -4.44)	17.8 MD, −5.7(−8.07 to −3.28)	<0.001	<0.001	N/A
	Moxifloxacin (n=666)	9.2 ± 5.3 MD, 2.26(1.15 to 3.38)	9.3 MD, 2.8 (1.4 to 4.2)	<0.001	<0.001	N/A
	Cefoperazone-sulbactam (n=241)	16.6 ± 9.6 MD, -5.18(-6.78 to -3.57 )	16.4 MD, −4.3 (−6.24 to −2.25)	<0.001	<0.001	N/A
Duration of antibiotics (days) MD (95%CI)	Omadacycline (n=177)	7.2 ± 3.0	7.2 (Reference)	N/A	N/A	N/A
	Tigecycline (n=124)	8.0 ± 4.4 MD, -0.76(-1.66 to 0.14)	8.0 MD, −0.9 (−1.78 to 0.05)	0.069	0.075	N/A
	Moxifloxacin (n=666)	6.5 ± 2.5 MD, 0.68(0.19 to1.17)	6.6 MD, 0.6 (0.39 to 1.68)	<0.001	<0.001	N/A
	Cefoperazone-sulbactam (n=241)	8.2 ± 4.8 MD, -1.00(-1.76 to -0.25)	8.2 MD, −1.1 (−1.94 to −0.39)	0.003	0.004	N/A
Microbiological clearance (%) RD (95%CI)	Omadacycline (n=177)	14/131 (10.7%)	8.7 (Reference)	N/A	N/A	N/A
	Tigecycline (n=124)	46/98 (46.9%) RD, −36.2(-46.7 to -25.7)	48.5 RD, −39.8 (−52.03 to −27.54)	<0.001	<0.001	N/A
	Moxifloxacin (n=666)	88/440 (20.0%) RD, −9.3(−15.9 to -2.7)	20.4 RD, −11.7(9.55 to 23.77)	<0.001	<0.001	N/A
	Cefoperazone-sulbactam (n=241)	33/159 (20.8%) RD, −10.1(−17.7 to -2.4)	21.0 RD, −12.3 (−21.76 to−5.48)	0.002	0.004	N/A

Data are presented after multinomial inverse probability of treatment weighting (IPTW) adjustment except for raw counts. RD, risk difference; MD, mean difference; CI, confidence interval; NNT, number needed to treat; FDR, false discovery rate. RD and MD values were calculated as omadacycline minus comparator. For binary outcomes, positive RD values indicate a higher rate in the omadacycline group than in the comparator group, whereas negative RD values indicate a lower rate in the omadacycline group. For continuous outcomes, negative MD values indicate shorter duration or lower values in the omadacycline group, whereas positive MD values indicate longer duration or higher values in the omadacycline group. Pairwise P-values were adjusted using the Benjamini-Hochberg FDR procedure. NNT was calculated only for statistically significant primary-outcome comparisons after FDR correction. The NNT of 21 favors omadacycline over moxifloxacin, whereas the NNT of 24 favors cefoperazone-sulbactam over omadacycline. Microbiological clearance analyses were exploratory because follow-up cultures were not systematically obtained for all patients. The microbiological denominators reflect the unweighted subset of patients with available follow-up cultures.

## 3 Results

### 3.1 Study population and baseline characteristics

This study initially identified 3,914 hospitalized patients aged ≥ 65 years with a confirmed diagnosis of community-acquired pneumonia (CAP) who were treated with omadacycline, tigecycline, moxifloxacin, or cefoperazone-sulbactam (**Figure 1**). After excluding patients who received antibiotic treatment for <72 hours (n=1,128) and those with non-bacterial pneumonia or other pulmonary diseases (n=1,578), the final analysis cohort comprised 1,208 patients, including 177 patients in the omadacycline group, 124 in the tigecycline group, 666 in the moxifloxacin group, and 241 in the cefoperazone-sulbactam group.

IPTW was applied to construct a weighted pseudo-population for comparative efficacy analysis. After weighting, the ESS values were 108.66 for the omadacycline group, 78.36 for the tigecycline group, 579.83 for the moxifloxacin group, and 167.19 for the cefoperazone-sulbactam group.

### 3.2 Covariate balance before and after weighting

The baseline demographic and clinical characteristics of the patient groups prior to adjustment for IPTW were detailed in **Table 2**. Most baseline variables showed significant differences, with maximum SMDs ranging from 0.122 to 0.845. Imbalances were particularly pronounced for the CURB-65 score (maximum SMD, 0.845) and COPD (maximum SMD, 0.540). These imbalances reflected non-random prescribing patterns in everyday clinical practice that require urgent attention.

**Table 2:** Baseline characteristics of the study population before IPTW.

Characteristic	Omadacycline(n=177)	Tigecycline (n=124)	Moxifloxacin (n=666)	Cefoperazone-sulbactam (n=241)	Maximum SMD
Age (median, IQR)	74(70,80)	72(69,78)	71(68,75)	72(69,78)	0.422
Height	163(156,170)	165(158,170)	165(158,170)	163(158,170)	-0.122
Weight	60(52,70)	65(55,75)	60(55,70)	60(52,70)	-0.405
BMI	23(20,26)	24(21,27)	23(21,25)	23(20,25)	0.338
CURB-65 score	1(1,2)	2(1,2)	1(1,2)	2(2,2)	-0.845
Gender (%)	98(55.4)	76(61.3)	360(54.1)	128(53.1)	0.166
Smoking (%)	65(36.7)	33(26.6)	263(39.5)	80(33.2)	-0.276
COPD (%)	11(6.2)	6(4.8)	152(22.8)	12(5.0)	-0.540

Data are presented as unweighted raw counts (percentages) for categorical variables and median (interquartile range) for continuous variables. CAP, community-acquired pneumonia; IPTW, inverse probability of treatment weighting; COPD, chronic obstructive pulmonary disease; CURB-65, confusion, urea, respiratory rate, blood pressure, and age ≥ 65 years score.

**Figure 2:**
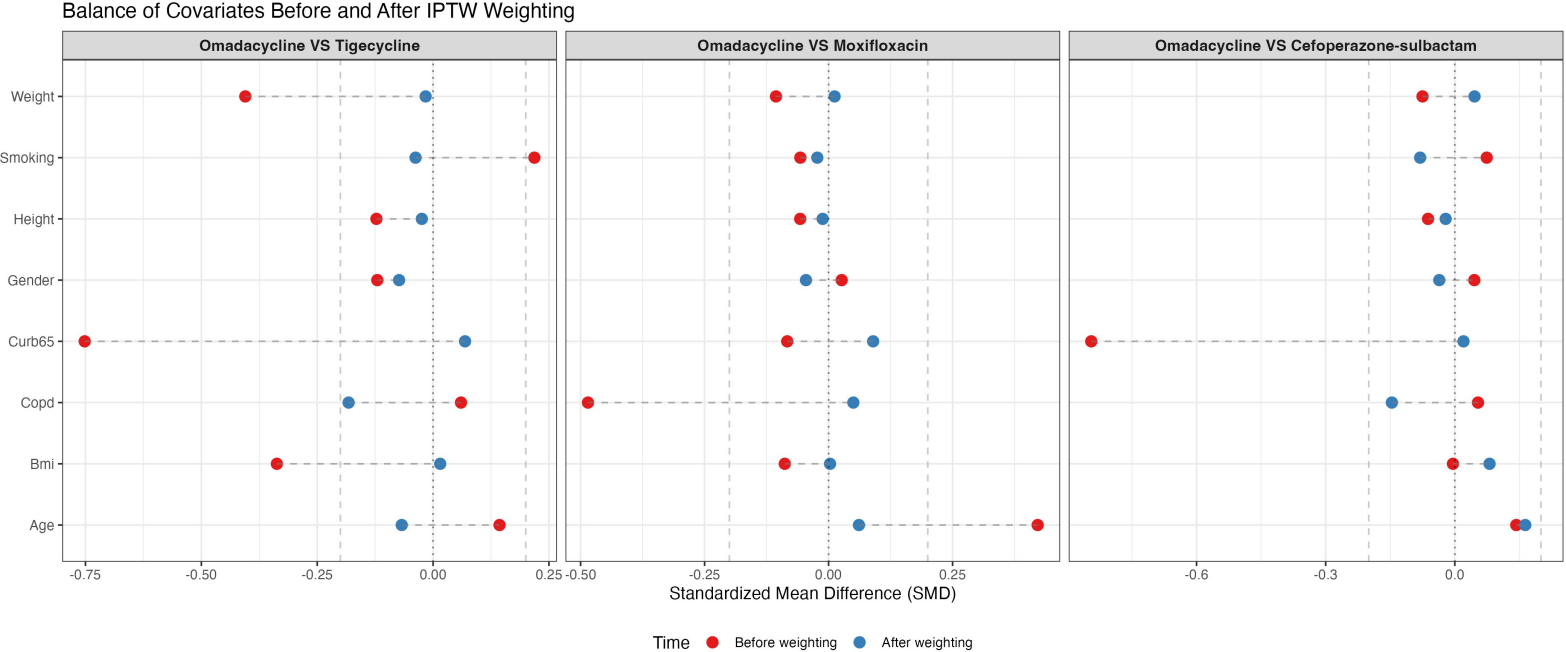
Love plot illustrating absolute standardized mean differences (SMD) for baseline covariates before and after multinomial IPTW adjustment. Description: This plot shows covariate balance across treatment groups before and after inverse probability of treatment weighting (IPTW). Red circles indicate unweighted absolute standardized mean differences (SMDs), and blue circles indicate IPTW-weighted absolute SMDs. Baseline covariates included age, height, weight, body mass index, CURB-65 score, sex, smoking history, and chronic obstructive pulmonary disease. Vertical dashed lines indicate the prespecified SMD threshold of 0.20. After IPTW adjustment, all assessed covariates had absolute SMDs below 0.20, indicating improved measured covariate balance across groups.

Following IPTW weighting, the maximum SMD for all baseline variables decreased significantly, with all SMDs values falling below 0.2 (**Table 3**, **Figure 2**). This indicated that the distribution of baseline variables across the treatment groups had been well balanced.

**Table 3:** Weighted baseline characteristics after IPTW adjustment.

Characteristic	Omadacycline (n=177)	Tigecycline (n=124)	Moxifloxacin (n=666)	Cefoperazone-sulbactam (n=241)	Maximum SMD
ESS	108.66	78.36	579.83	167.19	N/A
Age (median, IQR)	73(69,76)	73(69,78)	72(68,77)	71(68,77)	0.164
Height	165(156,170)	164(158,171)	165(158,170)	164(158,171)	-0.025
Weight	62(55,70)	60(50,70)	60(54,70)	60(50,71)	0.046
BMI	23(20,26)	23(20,25)	23(21,25)	23(21,25)	0.081
CURB-65 score	2(1,2)	1(1,2)	2(1,2)	2(1,2)	0.090
Gender (%)	43.61	47.26	45.87	45.41	-0.073
Smoking (%)	61.64	63.47	62.75	65.52	-0.081
COPD (%)	86.52	92.13	84.77	91.12	-0.182

IPTW, inverse probability of treatment weighting; SD, standard deviation; Ref, reference group. Maximum weight stabilization trimming was deployed at the 1st and 99th percentiles to restrict excessive variance inflation while preserving the structural integrity of the real-world sample size. The effective sample size (ESS) represents the design-effect-adjusted population headcount following the allocation of stabilized inverse probability weights, reflecting the true statistical power remaining for subsequent robust inference testing.

### 3.3 Outcomes before and after IPTW adjustment

The results for the primary and secondary outcome measures before and after adjustment for IPTW were shown in **Table 1**.

Regarding the primary effectiveness outcome, the unweighted pragmatic clinical response rates exceeded 93% across all treatment cohorts, indicating a substantial baseline ceiling effect. Prior to IPTW adjustment, the raw unadjusted risk differences (calculated as omadacycline minus the comparator) were −7.82% (95% CI, −14.55 to −1.09) for tigecycline, 2.83% (95% CI, −0.59 to 6.26) for moxifloxacin, and −1.97% (95% CI, −6.54 to 2.61) for cefoperazone-sulbactam.

After multinomial IPTW adjustment and Benjamini-Hochberg FDR correction, omadacycline showed a pragmatic clinical response comparable to tigecycline, higher than moxifloxacin, but lower than cefoperazone-sulbactam. The adjusted risk difference for omadacycline versus tigecycline was −0.80% (95% CI, −6.82 to 5.21; P = 0.782; q = 0.782), indicating no statistically significant difference. Compared with moxifloxacin, omadacycline was associated with a higher pragmatic clinical response rate, with an adjusted RD of 4.81% (95% CI, 2.63 to 6.98; P < 0.001; q = 0.044). In contrast, compared with cefoperazone-sulbactam, omadacycline was associated with a lower pragmatic clinical response rate, with an adjusted RD of −4.16% (95% CI, −8.29 to −0.03; P = 0.049; q = 0.044). These findings should be interpreted in the context of high baseline response rates and the broad pragmatic endpoint definition.

To quantify the absolute clinical relevance under the high baseline response rate, NNT was calculated for statistically significant primary-outcome comparisons after FDR correction. The adjusted comparison between omadacycline and moxifloxacin yielded an NNT of approximately 21 (95% CI, 14 to 38), indicating that approximately 21 elderly CAP patients would need to receive omadacycline rather than moxifloxacin to achieve one additional pragmatic clinical response. Conversely, the comparison with cefoperazone-sulbactam favored cefoperazone-sulbactam, with an NNT of approximately 24 (95% CI, 16 to 62), indicating that approximately 24 patients would need to receive cefoperazone-sulbactam rather than omadacycline to achieve one additional pragmatic clinical response. Because the comparison with tigecycline was not statistically significant, NNT was not calculated for this pair.

For secondary outcome, the IPTW-adjusted length of hospital stay differed significantly across comparator groups. Omadacycline was associated with a shorter hospital stay than tigecycline and cefoperazone-sulbactam, but a longer hospital stay than moxifloxacin. The adjusted MD was −5.7 days for tigecycline (95% CI, −8.07 to −3.28; P < 0.001; q < 0.001), 2.8 days for moxifloxacin (P < 0.001; q < 0.001), and −4.3 days for cefoperazone-sulbactam (95% CI, −6.24 to −2.25; P < 0.001; q < 0.001). These process outcomes should be interpreted cautiously because length of stay may be influenced by discharge procedures, prescribing preferences, and residual confounding.

Regarding antibiotic duration, omadacycline showed no statistically significant difference compared with tigecycline, but was associated with a longer duration than moxifloxacin and a shorter duration than cefoperazone-sulbactam. The adjusted MD was −0.9 days for tigecycline (95% CI, −1.78 to 0.05; P = 0.069; q = 0.075), 0.6 days for moxifloxacin (P < 0.001; q < 0.001), and −0.1 days for cefoperazone-sulbactam (P = 0.003; q = 0.004). Because antibiotic duration is strongly affected by clinical practice patterns and antimicrobial stewardship policies, these findings should be considered exploratory process indicators rather than direct treatment effects.

**Figure 3:**
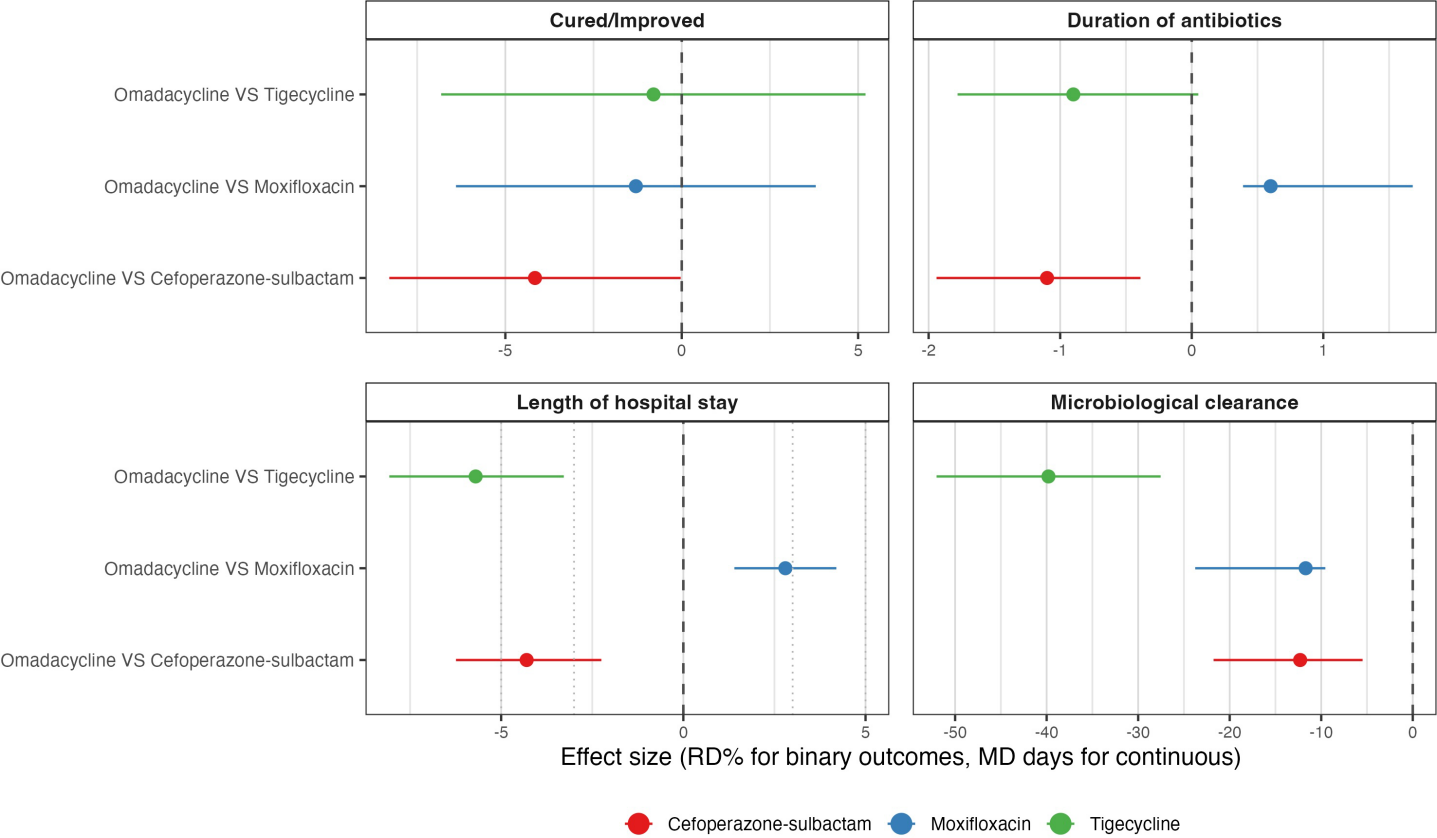
Forest plot of IPTW-adjusted risk differences and mean differences for primary and secondary outcomes. Description: This four-panel forest plot shows IPTW-adjusted treatment-effect estimates comparing omadacycline with tigecycline, moxifloxacin, and cefoperazone-sulbactam. The primary pragmatic clinical response and microbiological clearance are presented as adjusted risk differences (RDs), whereas hospital length of stay and antibiotic duration are presented as adjusted mean differences (MDs). Microbiological clearance analyses were restricted to patients with available follow-up microbiological data. Horizontal bars represent 95% confidence intervals, and the vertical dashed line indicates the null value of zero. Weighted estimates should be interpreted as adjusted associations rather than causal treatment effects.

Microbiological clearance was evaluated only among patients with available follow-up cultures and should therefore be interpreted as an exploratory analysis of a microbiologically evaluable subset. In this subset, weighted microbiological clearance was lower in the omadacycline group than in all three comparator groups. Compared with omadacycline, the adjusted RD was −39.8% for tigecycline, −11.7% for moxifloxacin, and −12.3% for cefoperazone-sulbactam. Because follow-up cultures were not routinely obtained and were likely influenced by clinical severity, persistent symptoms, or clinician concern for treatment failure, these microbiological findings should not be equated with overall comparative clinical effectiveness (**Figure 3**).

### 3.4 Sensitivity analysis

To evaluate the methodological robustness of the primary endpoint, a sensitivity analysis was conducted utilizing a strict three-point success criterion. This rigid definition required concurrent pre-discharge documentation of clinical, laboratory, and radiological improvement; unperformed examinations were automatically coded as non-responses. Under this strict framework, the adjusted models indicated that the pragmatic response rate for omadacycline was not significantly different from cefoperazone-sulbactam, but demonstrated a statistically lower association of success compared with tigecycline and moxifloxacin (**Table 4**).

**Table 4:** Sensitivity analysis with all three indicators valid.

Outcome	Omadacycline (n=177)	Tigecycline (n=124)	Moxifloxacin (n=666)	Cefoperazone-sulbactam (n=241)
Sensitivity outcome (all three effective) RD (95%CI)	3/174(1.7%)	11/113(9.7%) RD,−8.06(−15.05 to−1.07)	17/662(2.6%)RD,−2.43(−3.76 to−1.10)	3/225(1.3%) RD,0.82(−2.28 to 3.92)

IPTW, inverse probability of treatment weighting; OR, odds ratio; CI, confidence interval. The rigid success criterion enforced for this sensitivity framework strictly required concurrent and synchronized documentation of clinical symptom resolution, laboratory biomarker normalization, and radiological chest imaging improvement prior to hospital discharge. Patients lacking any single repeated diagnostic modality were automatically classified as treatment failures. Complete-case analyses utilized listwise deletion for missing post-treatment parameters. Due to the shorter mean length of stay in the omadacycline population, routine pre-discharge imaging was less frequently performed, resulting in mathematical separation and substantial power attenuation within this highly conservative sub-model. Interpretations must remain hypothesis-generating rather than definitive.

In the sensitivity analysis applying the strict three-point outcome definition (**Table 4**), omadacycline performed worse than its comparators. A data audit of pre-imputation records revealed that the post-treatment completion rates for clinical, laboratory, and radiological domains were 100.0%, 95.5%, and 75.7% in the omadacycline cohort, compared to 100.0%, 96.0%, and 85.5% in the tigecycline cohort (80.1% in the cefoperazone-sulbactam cohort, and 72.5% in the moxifloxacin cohort). Under the strict definition requiring simultaneous improvement in all three domains, unperformed examinations were coded as non-responses, reducing successful events in the unweighted omadacycline cohort to 3 cases. This induced extreme data sparsity and statistical instability under multinomial IPTW modeling.

To further assess the statistical consequences of missing data handling, a complete-case analysis was performed. Following the exclusion of patients with missing post-treatment parameters via listwise deletion, the restricted sub-cohorts comprised 110 cases for omadacycline, 60 for tigecycline, 499 for moxifloxacin, and 136 for cefoperazone-sulbactam. Within this complete-case framework, the IPTW-adjusted incidence of the primary outcome in the omadacycline group was 95.5%, which was not statistically significantly different from the tigecycline (96.7%), moxifloxacin (93.8%), or cefoperazone-sulbactam (97.1%) cohorts. While the non-significant comparison against tigecycline aligns with the primary model, the loss of statistical significance against moxifloxacin and cefoperazone-sulbactam highlights the methodological limitations of complete-case deletion. Systematically omitting patients lacking redundant post-treatment parameters fundamentally alters the baseline composition of the analyzed sample, typically excluding rapidly recovering individuals who were discharged early. This structural reduction in sample size substantially underpowered the adjusted comparative models, suggesting that the observed shifts in statistical significance may reflect both reduced statistical power and selection introduced by complete-case deletion. Therefore, the primary findings should be interpreted with caution (**Table 5**).

**Table 5:** Complete-case analysis with all covariates included.

Outcome	Omadacycline (n=177)	Tigecycline (n=124)	Moxifloxacin (n=666)	Cefoperazone-sulbactam (n=241)
Cured/Improved (%)	105/110 (95.5%)	58/60 (96.7%) RD,−2.41 (−8.86 to 4.03)	468/499 (93.8%) RD,1.07 (−4.00 to 6.14)	132/136 (97.1%) RD,-2.57 (−8.12 to 2.98)
Sensitivity outcome (all three effective) RD (95%CI)	3/110 (2.7%)	6/60 (10.0%) RD,−7.05 (−17.02 to 2.93)	16/499 (3.2%) RD,−0.00 (−4.25 to 4.24)	3/136 (2.2%) RD,1.59 (−3.63 to 6.81)

Data are presented after multinomial IPTW adjustment via listwise deletion. OR, odds ratio; CI, confidence interval. Reduced sample sizes reflect the restricted sub-cohorts after omitting individuals missing post-treatment metrics.

The E-value analysis supported cautious interpretation of the primary observational estimates. Although the point-estimate E-values were 3.24 for the omadacycline versus moxifloxacin comparison and 6.08 for the omadacycline versus cefoperazone-sulbactam comparison, the lower-bound E-values were 1.00 and 1.16, respectively. These results indicate that the confidence-limit estimates remain vulnerable to relatively weak unmeasured confounding, and the observed associations should not be interpreted causally.

### 3.5 Exploratory subgroup analysis

**Figure 4:**
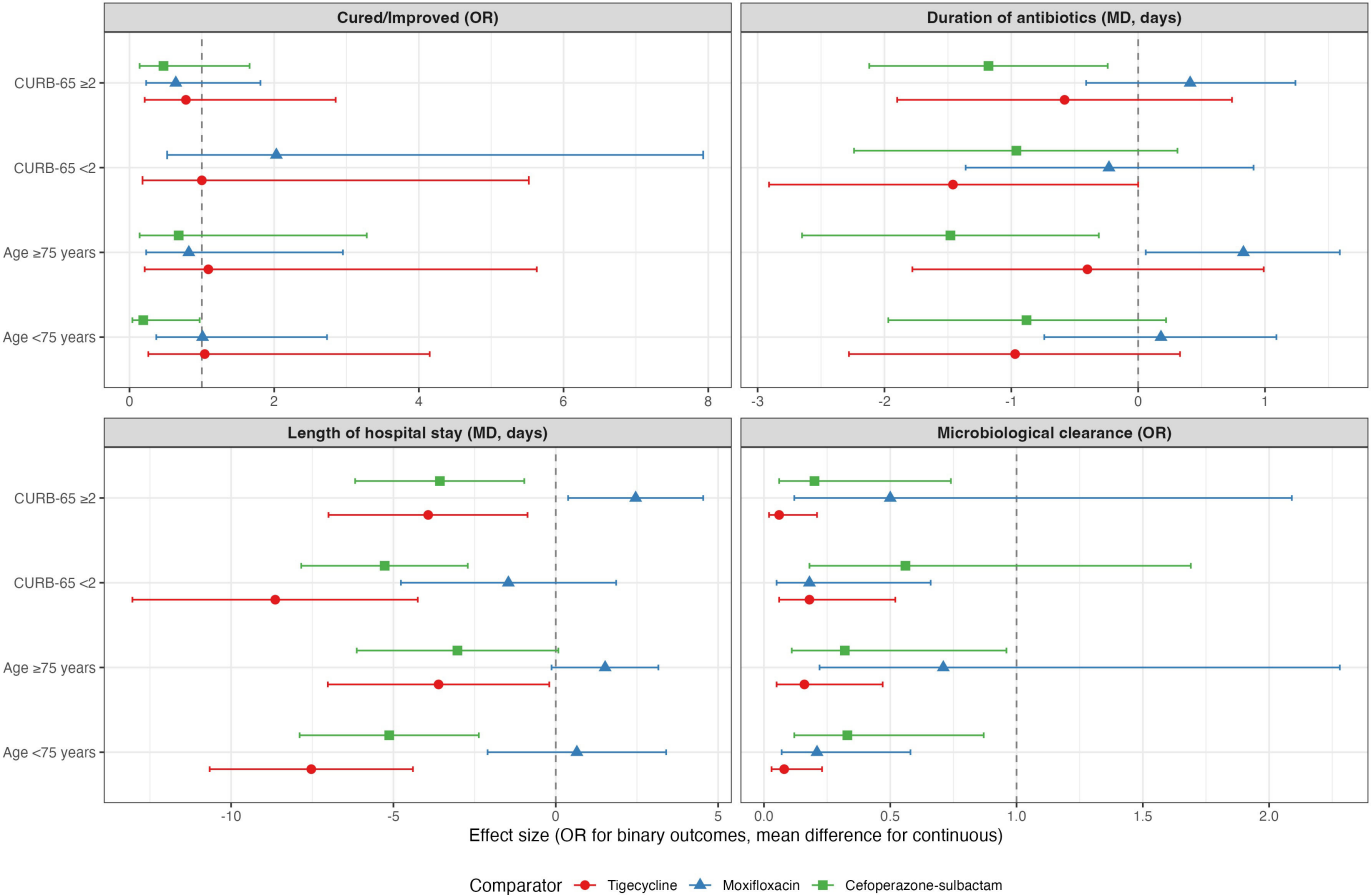
Stratified forest plots of exploratory subgroup analyses by age and disease severity. Description: This four-panel figure presents exploratory subgroup analyses comparing omadacycline with active control regimens according to age (<75 years vs ≥75 years) and disease severity (CURB-65 <2 vs ≥2). Treatment associations are shown as odds ratios (ORs) for binary outcomes and mean differences (MDs) for continuous outcomes. Vertical dashed reference lines indicate the null values of 1.0 for ORs and 0 for MDs. Subgroup analyses were exploratory, involved reduced effective sample sizes within strata, and were not intended to support treatment-selection strategies. Interaction P-values were not adjusted for multiplicity and should be interpreted as hypothesis-generating.

To explore the potential consistency of the primary findings across distinct clinical phenotypes, exploratory subgroup analyses were performed stratified by age (<75 years vs. ≥75 years) and baseline disease severity (CURB-65 score <2 vs. ≥2). All stratified evaluation metrics within these sub-models were explicitly classified *a priori* as exploratory and hypothesis-generating only, given the substantial statistical power attenuation driven by stratum-specific sample fragmentation (**Figure 4**; **Table 6**).

**Table 6:** Exploratory subgroup analysis of pragmatic clinical response stratified by age and severity demographics.

Outcome	Subgroup	Omadacycline	Tigecycline	P	Moxifloxacin	P	Cefoperazone-sulbactam	P
Cured/Improved (%) (OR,95%CI)	Age<75 years	Reference	1.04 (0.26 to 4.15)	0.959	1.01 (0.37 to 2.73)	0.982	0.19 (0.04 to 0.97)	0.046
	Age≥75 years	Reference	1.09 (0.21to 5.63)	0.915	0.82 (0.23 to 2.95)	0.760	0.68 (0.14 to 3.28)	0.633
	CURB-65<2	Reference	1.00 (0.18 to 5.52)	>0.999	2.03 (0.52 to 7.93)	0.310	0.00 (0.00 to 0.00)	<0.001
	CURB-65≥2	Reference	0.78 (0.21 to 2.85)	0.703	0.64 (0.23 to 1.81)	0.401	0.47 (0.14 to 1.66)	0.242
Length of hospital stay (days) (MD,95%CI)	Age<75 years	Reference	−7.53 (-10.66 to −4.40)	<0.001	0.65 (-2.10 to 3.40)	0.641	−5.13 (−7.89 to−2.37)	<0.001
	Age≥75 years	Reference	−3.61 (−7.02 to −0.20)	0.038	1.52 (-0.13 to 3.16)	0.071	−3.03 (−6.13 to 0.08)	0.056
	CURB-65<2	Reference	−8.64 (−13.04 to −4.25)	<0.001	−1.46 (−4.77 to 1.86)	0.389	−5.27 (−7.84 to -2.71)	<0.001
	CURB-65≥2	Reference	−3.93 (−7.00 to −0.87)	0.012	2.46 (0.38 to 4.54)	0.020	−3.57 (−6.18 to −0.97)	0.007
Duration of antibiotics (days) (MD,95%CI)	Age<75 years	Reference	-0.97 (-2.28 to 0.33)	0.145	0.18 (-0.74 to 1.09)	0.700	-0.88 (-1.97 to 0.22)	0.116
	Age≥75 years	Reference	-0.40 (-1.78 to 0.99)	0.575	0.83 (0.06 to 1.59)	0.034	-1.48 (-2.65 to -0.31)	0.013
	CURB-65<2	Reference	−1.46 (−2.91 to −0.00)	0.049	-0.23 (−1.36 to 0.91)	0.696	−0.96 (−2.24 to 0.31)	0.139
	CURB-65≥2	Reference	−0.58 (−1.90 to 0.74)	0.387	0.41 (−0.41 to 1.24)	0.328	−1.18 (−2.12 to −0.24)	0.014
Microbiological clearance (OR,95%CI)	Age<75 years	Reference	0.08 (0.03 to 0.23)	<0.001	0.21 (0.07 to 0.58)	0.003	0.33 (0.12 to 0.87)	0.026
	Age≥75 years	Reference	0.16 (0.05 to 0.47)	<0.001	0.71 (0.22 to 2.28)	0.567	0.32 (0.11 to 0.96)	0.042
	CURB-65<2	Reference	0.18 (0.06 to 0.52)	0.002	0.18 (0.05 to 0.66)	0.010	0.56 (0.18 to 1.69)	0.301
	CURB-65≥2	Reference	0.06 (0.02 to 0.21)	<0.001	0.50 (0.12 to 2.09)	0.341	0.20 (0.06 to 0.74)	0.016

IPTW, inverse probability of treatment weighting; OR, odds ratio; CI, confidence interval; CURB-65, confusion, urea, respiratory rate, blood pressure, and age ≥ 65 years score. Subgroup models partitioned by a CURB-65 score threshold of 2 are intended strictly for exploratory profiling and hypothesis generation. Model specifications utilize the robust sandwich variance estimator to protect against residual intra-cluster correlations. P-values for interaction evaluate whether the comparative efficacy of omadacycline varies significantly across low-to-moderate versus high baseline severity spectra. Several interaction terms reached nominal statistical significance; however, these exploratory findings should be interpreted cautiously because of low event rates, multiple comparisons, and reduced effective sample sizes within subgroups. For microbiological subgroup models, the dependent variable was microbiological non-clearance/persistence. Therefore, OR<1 indicates lower odds of persistence, indirectly reflecting higher microbiological clearance compared with omadacycline.

Regarding primary outcomes: Subgroup logistic models used non-response as the dependent variable (failure to meet pragmatic response). Non-response rates were low (0.0% to 5.63%) across subgroups. Compared with tigecycline and moxifloxacin, omadacycline showed no significant differences in the incidence of the primary outcome across all subgroups (all P > 0.05). However, in the subgroups aged <75 years (OR=0.19, P=0.046) and CURB-65 < 2 (OR=0.00, P < 0.001), omadacycline showed a significant difference compared with cefoperazone-sulbactam. In the remaining subgroups, no significant differences were observed between the two groups.

Regarding length of hospital stay: In all subgroups, omadacycline was associated with a significantly shorter hospital stay than tigecycline and cefoperazone-sulbactam (all P < 0.05), except for the ≥ 75-year subgroup comparison with cefoperazone-sulbactam (P=0.056). Compared with moxifloxacin, no significant differences in hospital stay were observed in the < 75-year, ≥75-year, and CURB-65<2 subgroups (all P>0.05). However, in the CURB-65 ≥ 2 subgroup, omadacycline was associated with a significantly longer hospital stay than moxifloxacin (MD=2.46, 95%CI, 0.38 to 4.54, P=0.020).

Regarding the duration of antibiotic: In the subgroup of patients aged <75 years, there was no significant difference in the duration of antibiotic treatment between omadacycline and any of the control groups (P > 0.05). In the subgroup of patients aged ≥ 75 years, the duration of antibiotic treatment with omadacycline was significantly longer than that with moxifloxacin (MD = 0.83, 95% CI, 0.06 to 1.59, P=0.034), but was significantly shorter than that of cefoperazone-sulbactam (MD=−1.48, 95% CI, −2.65 to −0.31, P=0.013). No significant difference was observed compared with tigecycline. In the CURB-65<2 subgroup, the duration of antibiotic treatment with omadacycline was significantly shorter than that with tigecycline (MD=−1.46, 95% CI −2.91 to −0.00, P=0.049), with no significant difference compared with moxifloxacin or cefoperazone-sulbactam. In the CURB-65≥2 subgroup, the duration of antibiotic treatment with omadacycline was significantly shorter than that with cefoperazone-sulbactam (MD=−1.18, 95%CI −2.12 to −0.24, P=0.014), but there was no significant difference compared with the other two groups.

Regarding microbiological non-clearance (persistence): In subgroup logistic models, the dependent variable was microbiological non-clearance among patients with follow-up cultures; OR<1 indicates a lower risk of persistence (i.e., higher clearance) compared with omadacycline. Tigecycline was associated with significantly lower odds of persistence than omadacycline in all subgroups (OR range 0.06–0.18, all P<0.01). Moxifloxacin was associated with lower odds of persistence than omadacycline in the <75 years age subgroup (OR=0.21, P=0.003) and the CURB-65<2 subgroup (OR=0.18, P=0.01), with no significant differences observed in the remaining subgroups. Cefoperazone-sulbactam was associated with lower odds of persistence than omadacycline in the <75 years (OR=0.33, P=0.026), ≥75 years (OR=0.32, P=0.042), and CURB-65≥2 (OR=0.20, P=0.016) subgroups, with no significant difference in the CURB-65<2 subgroup.

### 3.6 Interaction test

To assess whether treatment efficacy was influenced by age or the CURB-65 score, we included interaction terms between the treatment group and the subgroup variables in a weighted regression model and calculated the P-values using the Wald test. The results were summarized in **Table 7**.

**Table 7:** Interaction tests in subgroup analysis.

Outcome	Subgroup	Comparison	P
Cured/Improved (%)	Age	Omadacycline vs Tigecycline ≥ 75 years	0.976
		Omadacycline vs Moxifloxacin ≥ 75 years	0.024
		Omadacycline vs Cefoperazone-sulbactam ≥ 75 years	0.292
	CURB-65	Omadacycline vs Tigecycline ≥ 2	0.928
		Omadacycline vs Moxifloxacin ≥ 2	0.004
		Omadacycline vs Cefoperazone-sulbactam ≥ 2	<0.001
Length of hospital stay (days)	Age	Omadacycline vs Tigecycline ≥ 75 years	0.085
		Omadacycline vs Moxifloxacin ≥ 75 years	<0.001
		Omadacycline vs Cefoperazone-sulbactam ≥ 75 years	0.120
	CURB-65	Omadacycline vs Tigecycline ≥ 2	0.092
		Omadacycline vs Moxifloxacin ≥ 2	<0.001
		Omadacycline vs Cefoperazone-sulbactam ≥ 2	0.171
Duration of antibiotics (days)	Age	Omadacycline vs Tigecycline ≥ 75 years	0.832
		Omadacycline vs Moxifloxacin ≥ 75 years	<0.001
		Omadacycline vs Cefoperazone-sulbactam ≥75 years	0.513
	CURB-65	Omadacycline vs Tigecycline ≥ 2	0.400
		Omadacycline vs Moxifloxacin ≥ 2	<0.001
		Omadacycline vs Cefoperazone-sulbactam ≥ 2	0.889
Microbiological clearance (%)	Age	Omadacycline vs Tigecycline ≥ 75 years	0.651
		Omadacycline vs Moxifloxacin ≥ 75 years	0.002
		Omadacycline vs Cefoperazone-sulbactam ≥ 75 years	0.781
	CURB-65	Omadacycline vs Tigecycline ≥ 2	0.238
		Omadacycline vs Moxifloxacin ≥ 2	0.014
		Omadacycline vs Cefoperazone-sulbactam ≥ 2	0.122

MD, mean difference; OR, odds ratio; CI, confidence interval. Interaction P-values were not adjusted for multiplicity and should be interpreted as exploratory and hypothesis-generating.

An analysis of interactions in the clinical cure response rate revealed that, among the interactions between age and tigecycline (P=0.976), moxifloxacin (P=0.024) and cefoperazone-sulbactam (P=0.292), only the interaction term between moxifloxacin and age was statistically significant. This suggested that, compared with omadacycline, age modulated the clinical association of moxifloxacin. Interactions between CURB-65 and tigecycline (P = 0.928), moxifloxacin (P = 0.004), and cefoperazone-sulbactam (P < 0.001) were analyzed. The results indicated that there was a significant interaction between moxifloxacin and cefoperazone-sulbactam in terms of the CURB-65 score. This suggested that the CURB-65 score influenced the differences in efficacy between these two drug groups and omadacycline. However, given the low incidence of the primary outcome, the clinical significance of these interactions should be interpreted with caution.

With regard to the length of hospital days: there was a significant interaction between age and moxifloxacin (P<0.001), but no significant interaction with tigecycline (P=0.085) or cefoperazone-sulbactam (P=0.120). There was a significant interaction between the CURB-65 score and moxifloxacin (P<0.001), but no significant interaction with tigecycline (P=0.092) or cefoperazone-sulbactam (P=0.171). This suggested that the advantage of moxifloxacin in reducing length of hospital stay varies across different age and CURB-65 subgroups.

With regard to the duration of antibiotic treatment, there was a significant interaction between age and moxifloxacin in the omadacycline group (P < 0.001). No significant interaction was observed with tigecycline (P = 0.832) or cefoperazone-sulbactam (P = 0.513). There was a significant interaction between CURB-65 and moxifloxacin (P<0.001), but no significant interaction with tigecycline (P=0.400) or cefoperazone-sulbactam (P=0.889).

With regard to microbial clearance, there was a significant interaction between age in the omadacycline group and moxifloxacin (P=0.002), but no significant interaction with tigecycline (P=0.651) or cefoperazone-sulbactam (P=0.781). There was a significant interaction between CURB-65 and moxifloxacin (P=0.014), but no significant interaction with tigecycline (P=0.238) or cefoperazone-sulbactam (P=0.122).

### 3.7 Safety outcomes

After adjustment for IPTW, the incidence of adverse events in the omadacycline group was 0.56%. This was significantly lower than the 6.45% observed in the tigecycline group (RD = −7.37%, 95% CI, −13.49% to −1.25%, P = 0.018). It was also significantly lower than that in the moxifloxacin group (RD = −1.78%, 95% CI, −2.98% to −0.57%, P = 0.004). Compared with the cefoperazone-sulbactam group (3.73%), the difference was not statistically significant (RD = −3.31%, 95% CI, −6.64% to 0.02%, P = 0.052). The weighted incidence rates of adverse events and comparisons between groups were shown in **Table 8**.

**Table 8:** Safety analysis.

Outcome	Omadacycline (n=177)	Tigecycline (n=124)	Moxifloxacin (n=666)	Cefoperazone-sulbactam (n=241)
Adverse events(%)	1(0.56)	8 (6.45)	7 (1.05)	9 (3.73)
RD (95%CI)	Reference	−7.37 (−13.49 to −1.25)	−1.78 (−2.98 to −0.57)	−3.31 (−6.64 to 0.02)
P		0.018	0.004	0.052

Data denote raw unweighted event frequencies (percentages) alongside adjusted risks. AE, adverse event; CTCAE, Common Terminology Criteria for Adverse Events. Adverse event ascertainment was executed via a structured retrospective electronic medical record audit scanning standardized safety keywords, laboratory critical values (e.g., severe hepatic or renal enzyme excursions), and free-text clinician progress entries recorded throughout the active antibiotic deployment window. All documented toxicities were clinically adjudicated and graded in strict accordance with the Common Terminology Criteria for Adverse Events (CTCAE, version 5.0). Events categorized as drug-related reflect the consensus agreement of two independent blinded clinical review investigators.

In the primary IPTW-weighted safety analysis, the incidence of recorded adverse events was lowest in the omadacycline group. Omadacycline was associated with fewer adverse events than tigecycline and moxifloxacin, whereas the comparison with cefoperazone-sulbactam did not reach statistical significance in the primary weighted analysis. Given the low absolute event counts, supplementary safety sensitivity analyses were performed using unweighted Fisher’s exact tests and Firth-penalized logistic regression. These analyses consistently showed lower odds of adverse events with omadacycline than with tigecycline. The comparison with moxifloxacin was no longer statistically significant in the Fisher and Firth analyses, whereas the comparison with cefoperazone-sulbactam became statistically significant. Therefore, the safety findings suggest a generally favorable tolerability pattern for omadacycline, but comparisons beyond tigecycline should be interpreted cautiously because of rare-event sparsity and model dependence.

### 3.8 Analysis of all-cause mortality

Regarding survival metrics, the crude unweighted 30-day all-cause mortality rates were 1.69% (3/177) in the omadacycline cohort, 8.87% (11/124) in the tigecycline cohort, 0.60% (4/666) in the moxifloxacin cohort, and 6.64% (16/241) in the cefoperazone-sulbactam cohort (**Table 9**). Following multinomial IPTW adjustment, the adjusted GEE logistic regression analysis revealed no statistically significant differences in the odds of 30-day all-cause mortality between the omadacycline cohort (Reference) and moxifloxacin (adjusted OR = 1.61; 95% CI, 0.34 to 7.53; P = 0.545), tigecycline (adjusted OR = 0.81; 95% CI, 0.62 to 1.06; P = 0.125), or cefoperazone-sulbactam (adjusted OR = 1.11; 95% CI, 0.89 to 1.40; P = 0.355). No statistically significant difference in 30-day all-cause mortality was observed after weight trimming and GEE modeling. However, given the small number of deaths and potential residual confounding from unmeasured goals-of-care factors, these findings should be interpreted as exploratory (**Table 9**).

**Table 9:** Mortality analysis.

	Omadacycline(n=177)	Tigecycline (n=124)	Moxifloxacin (n=666)	Cefoperazone-sulbactam (n=241)
Mortality rate (%) OR (95%CI)	3 (1.69) Reference	11 (8.87) 0.81 (0.62 to 1.06)	4 (0.60) 1.61 (0.34 to 7.53)	16 (6.64) 1.11 (0.89 to 1.4)
P		0.125	0.545	0.355

Odds ratios for 30-day mortality were estimated using GEE logistic models after implementing a standard 1% and 99% weight trimming protocol to control for extreme numerical instability from rare-event separation.

## 4 Discussion

The main findings of this study should be interpreted as observational associations rather than causal treatment effects. After IPTW adjustment and FDR correction, omadacycline showed a pragmatic clinical response comparable to tigecycline, higher than moxifloxacin, but lower than cefoperazone-sulbactam. The absolute differences were modest under a high baseline response rate, with an NNT of approximately 21 favoring omadacycline over moxifloxacin and an NNT of approximately 24 favoring cefoperazone-sulbactam over omadacycline. Regarding process outcomes, omadacycline was associated with shorter hospital stay than tigecycline and cefoperazone-sulbactam, but longer hospital stay than moxifloxacin. Antibiotic duration was comparable to tigecycline, shorter than cefoperazone-sulbactam, but longer than moxifloxacin. Microbiological clearance was lower in the omadacycline group than in all comparator groups within the microbiologically evaluable subset. Recorded adverse events were fewer with omadacycline in the primary weighted analysis, although rare-event sensitivity analyses indicated that safety comparisons should be interpreted cautiously.

The findings of this study should also be interpreted in the context of existing prospective and real-world evidence. The pivotal OPTIC trial has demonstrated that omadacycline is non-inferior to moxifloxacin in the treatment of CAP, with similar overall safety profiles (Lodise et al., 2021). A randomised controlled trial published by Yu in 2025 involving elderly CAP patients showed that the clinical response rate in the omadacycline group was significantly higher than that in the moxifloxacin group, consistent with the findings of this study indicating the superiority of omadacycline over moxifloxacin (Yu et al., 2025). Our study extends prior evidence by evaluating omadacycline in a real-world elderly hospitalized cohort and by including comparator regimens commonly used in routine clinical practice, including tigecycline and cefoperazone-sulbactam. However, direct comparisons across studies should be made cautiously because of differences in study design, patient populations, disease severity, endpoint definitions, follow-up procedures, and comparator regimens.

Regarding cefoperazone-sulbactam, a multicenter study by Huang involving 941 elderly patients with pneumonia demonstrated that cefoperazone-sulbactam was an effective alternative to piperacillin/tazobactam as empirical therapy, with a pragmatic clinical response of 81%–83% (Huang et al., 2022). The study by Xia further confirmed that the 30-day survival rate among elderly patients with CRAB pneumonia treated with cefoperazone-sulbactam was significantly higher than that of patients who did not receive the treatment (Xia et al., 2014). These findings provide relevant clinical context for the observed performance of cefoperazone-sulbactam in the present study. Nevertheless, differences in infection type, pathogen distribution, disease severity, and study design limit direct comparison with our cohort.

It is worth noting that the clinical response rates were generally high across all four groups, exceeding 93%. This suggests that the primary outcome measures used in this study may exhibit a marked”ceiling effect”. Given the high pragmatic clinical response rates across all groups, the statistically significant differences in the primary outcome should be interpreted in terms of absolute effect size. The NNT for omadacycline versus moxifloxacin was approximately 21, suggesting a modest absolute advantage of omadacycline over moxifloxacin. By contrast, the comparison with cefoperazone-sulbactam showed the opposite direction of association: cefoperazone-sulbactam had a higher pragmatic clinical response rate than omadacycline, corresponding to an NNT of approximately 24 in favor of cefoperazone-sulbactam. Although statistically significant, these absolute differences were modest and should be interpreted in the context of the substantial ceiling effect associated with the primary endpoint. Therefore, these NNT values do not uniformly support an advantage of omadacycline across all comparators; rather, they indicate small absolute differences under a high baseline response rate. Overall, even when statistically significant, the observed differences in pragmatic clinical response should be interpreted as modest in clinical magnitude. The comparison with moxifloxacin deserves careful interpretation because the direction of association differed across endpoint types. Omadacycline was associated with a higher pragmatic clinical response rate than moxifloxacin, but with longer length of hospital stay and longer antibiotic duration. This pattern suggests that the pragmatic clinical response endpoint and process outcomes may capture different dimensions of real-world care. The broad endpoint definition may have inflated treatment success rates because improvement in only one domain was sufficient to classify a patient as a responder. Consequently, the observed response rates exceeding 93% across treatment groups should not be interpreted as equivalent to standardized clinical cure rates or comprehensive clinical recovery. Length of stay and antibiotic duration are influenced not only by clinical recovery but also by prescribing habits, discharge logistics, route of administration, sequential therapy decisions, and institutional antimicrobial stewardship practices. Therefore, the apparent advantage in pragmatic response should not be interpreted as a uniform advantage across all clinical and process outcomes.

In this study, cefoperazone-sulbactam showed the highest pragmatic clinical response rate. This result is consistent with the dominant position of β-lactam antibiotics in the empirical treatment of CAP, particularly when targeting typical bacterial infections (Arnold, 2017). In contrast, the observed association between omadacycline and a higher pragmatic clinical response rate than moxifloxacin may be related to the baseline characteristics of patients in the moxifloxacin group in this study. Among elderly CAP patients, the proportion of Enterobacteriaceae and Pseudomonas species is typically higher (Sahuquillo-Arce et al., 2016). Furthermore, regional variations in the resistance landscape may also influence the actual efficacy of the drugs. Arnold’s review notes that for elderly CAP patients requiring hospitalisation, empirical antimicrobial regimens should cover both typical and atypical pathogens (Arnold, 2017). Recommended treatment strategies include the combination of a β-lactam with a macrolide, or the use of a respiratory fluoroquinolone as monotherapy. The superior performance of cefoperazone-sulbactam in this study is consistent with this treatment framework. Cefoperazone-sulbactam showed a higher pragmatic clinical response rate than omadacycline, corresponding to an NNT of approximately 24 in favor of cefoperazone-sulbactam. However, omadacycline was associated with shorter length of stay and shorter antibiotic duration than cefoperazone-sulbactam. These findings should not be interpreted as demonstrating overall superiority of one regimen over another, because clinical response, length of stay, and antibiotic duration may be affected by different clinical and non-clinical factors.

Differences in length of stay and antibiotic duration may have clinical relevance, but these process outcomes should be interpreted cautiously, particularly in elderly patients requiring simplified treatment pathways. However, these process outcomes are susceptible to residual confounding, discharge practices, prescribing preferences, and antimicrobial stewardship policies, and therefore should not be interpreted as direct treatment effects. From a pharmacokinetic perspective, omadacycline possesses several favourable characteristics. Its absolute bioavailability reaches 34.5% and an oral dose of 300 mg achieves equivalent exposure levels to an intravenous dose of 100 mg (Rodvold and Pai, 2019). This characteristic provides the pharmacological basis for sequential intravenous-oral therapy. With regard to drug interactions, the metabolism of omadacycline does not rely on UDP-glucuronosyltransferase or the cytochrome P450 enzyme system (Sun et al., 2025). This implies a lower risk of interactions with medications commonly used by elderly patients (Berg et al., 2018). This characteristic is of particular value in the elderly population, who often suffer from multiple co-morbidities and require polypharmacy.

However, as process outcomes, length of stay and duration of antibiotic treatment are susceptible to non-clinical factors. The additional time patients spend in hospital after meeting discharge criteria may be determined by factors such as bed turnover, discharge procedures and social factors (Harhay et al., 2017). Furthermore, prescribing practices and departmental antimicrobial stewardship policies may also influence the duration of treatment (Ma et al., 2026). Consequently, conclusions regarding advantages in process outcomes should be interpreted with caution,as residual confounding or process bias cannot be ruled out.

The microbiological findings require particularly cautious interpretation. In the microbiologically evaluable subset, omadacycline showed lower microbiological clearance than tigecycline, moxifloxacin, and cefoperazone-sulbactam. However, follow-up cultures were not obtained according to a standardized protocol and were available only in a selected subset of patients. Repeat cultures may have been more likely among patients with persistent symptoms, severe disease, suspected treatment failure, colonization, or clinician concern for resistant pathogens. Therefore, selection bias cannot be excluded. Microbiological clearance in this study should be regarded as an exploratory and descriptive pathogen-related endpoint rather than a definitive surrogate for overall comparative clinical effectiveness. The discordance between pragmatic clinical response and microbiological clearance may reflect differences between the clinically evaluable cohort and the microbiologically evaluable subset, selective culture practices, persistent colonization, or residual confounding, rather than true differences in comparative clinical efficacy. Therefore, comparisons of microbiological clearance across treatment groups should be interpreted cautiously. Future studies should standardize microbiological sampling and clearly report culture follow-up mechanisms to enable reliable assessment of pathogen-level outcomes.

Subgroup analysis revealed heterogeneity in treatment response among elderly CAP patients based on age and disease severity (Yu et al., 2025). In patients aged <75 years or with a CURB-65 score <2, cefoperazone-sulbactam showed a higher pragmatic clinical response than omadacycline in the subgroup analysis. However, this observation should not be interpreted as evidence that β-lactam antibiotics are more suitable for mild elderly patients, because the subgroup analysis was exploratory and may have been affected by multiple comparisons, reduced effective sample sizes, and residual confounding. These results are consistent with clinical findings: a higher proportion of typical bacterial infections are observed in mild elderly patients and cefoperazone-sulbactam, as a broad-spectrum β-lactam antibiotic, provides effective coverage against common pathogens (Womack and Kropa, 2022). In contrast, among patients aged≥75 years or with a CURB-65 score≥2, the differences in efficacy between treatment groups narrowed. Omadacycline showed favorable associations with process outcomes and recorded adverse events in some elderly or more severe subgroups. However, because these analyses were exploratory and potentially affected by residual confounding and reduced effective sample sizes, they should not be interpreted as evidence that omadacycline is the preferred regimen. As elderly patients often require multiple medications and may experience reduced organ function, omadacycline’s lack of need for dose adjustment and limited potential for drug interactions remain pharmacological features that may be clinically relevant in this population (Corsonello et al., 2015). However, the sample size for subgroup analyses was limited, particularly in the CURB-65≥2 subgroup, leading to reduced estimation precision. Although several interaction terms reached nominal statistical significance, these subgroup findings should be interpreted cautiously because of low event rates, multiple comparisons, and reduced effective sample sizes within strata. Therefore, the above results should be regarded as exploratory and hypothesis-generating in nature. These findings are intended to provide insights for future research rather than serving as direct evidence for clinical decision-making.

The sensitivity analyses yielded findings that were not fully consistent with the primary analysis, indicating that the estimated associations were sensitive to endpoint definition and missing-data handling. Under the strict three-domain criterion, treatment success required simultaneous documentation of clinical, laboratory, and radiological improvement before discharge. In a retrospective real-world setting, this definition is highly dependent on whether repeat laboratory tests and follow-up imaging were performed. The data audit showed that radiological follow-up was incomplete and differed across treatment groups, and the omadacycline group had a shorter observed length of stay than several comparator groups. Because radiological resolution of pneumonia may lag behind symptomatic and biomarker improvement, pre-discharge imaging may not fully capture clinical recovery. These factors may partly explain the discordance between the primary and strict sensitivity analyses. However, the possibility of true endpoint instability cannot be excluded. This finding indicates that treatment-effect estimates may vary depending on whether the endpoint is defined as pragmatic improvement in any one domain or as simultaneous improvement across all three domains. Therefore, the primary outcome should be interpreted as a pragmatic improvement measure rather than a definitive clinical cure endpoint, and the comparative findings should be considered exploratory pending prospective validation using standardized CAP endpoints such as early clinical response, time to clinical stability, readmission, and mortality.

The safety analysis suggested a generally favorable tolerability profile for omadacycline, but the findings should be interpreted in the context of rare events. In the primary weighted analysis, recorded adverse events were less frequent in the omadacycline group than in the tigecycline and moxifloxacin groups. However, Fisher’s exact test and Firth-penalized logistic regression showed that the comparison with moxifloxacin was not statistically significant, whereas the comparisons with tigecycline and cefoperazone-sulbactam showed lower odds of adverse events with omadacycline. These discrepancies indicate that rare-event sparsity and model choice influenced the statistical interpretation. Therefore, the safety findings should be considered supportive but exploratory.

Similarly, the 30-day all-cause mortality analysis demonstrated no statistically significant differences between omadacycline and any of the active comparators after stabilizing outlier weights. Although the raw mortality numbers were structurally small, inflating the standard errors and widening the confidence intervals (such as the moxifloxacin comparison), these findings indicate that the choice of omadacycline was not associated with a differential survival risk. Given the observational constraints and potential for residual confounding from unmeasured baseline terminal frailty or documented goals-of-care limitations, these mortality evaluations should be interpreted strictly as exploratory associations rather than definitive causal evidence. Future prospective investigations utilizing structured geriatric assessments are necessary to fully elucidate the comparative survival profiles of these anti-infective regimens.

This study has several strengths. Firstly, this study was based on real-world data and involves 1,208 elderly patients with CAP. The relatively large sample size allowed for a comprehensive reflection of clinical practice. Secondly, the study compared omadacycline with three treatment regimens: tigecycline, moxifloxacin, and cefoperazone-sulbactam. This comparison addressed an evidence gap in the evidence regarding head−to−head evaluations of these drugs in the elderly population. The use of IPTW effectively balanced baseline covariates between groups, whilst multiple imputation methods were employed to handle missing data, thereby enhancing the accuracy of the estimates. Furthermore, the robustness of the results was validated through complete case analysis, strict outcome definitions and various sensitivity analyses, including Fisher and Firth regression.

Taken together, these design features strengthen the interpretability of the findings; however, several important limitations related to the retrospective single-center design, residual confounding, broad endpoint definition, incomplete microbiological follow-up, exploratory subgroup analyses, and sparse mortality or safety events should also be considered.

Several limitations should be emphasized. First, although IPTW improved the balance of measured baseline covariates, residual and unmeasured confounding remains unavoidable in this single-center retrospective study. Important prognostic factors, including frailty, baseline functional status, DNR/DNI orders, prior antibiotic exposure, aspiration risk, microbiological severity, pathogen burden, and treatment intent, were not consistently available in structured electronic records and could not be incorporated into the propensity score model. The E-value analysis was added as a quantitative sensitivity analysis, but it does not eliminate residual confounding. The lower-bound E-values close to 1.00 indicate that the observed associations remain vulnerable to relatively weak unmeasured confounding. Consequently, the observed associations should not be interpreted as causal treatment effects, but rather as exploratory observational findings that require confirmation in prospective studies with more comprehensive adjustment for potential confounders.

Second, the primary endpoint was a broad pragmatic improvement measure rather than a standardized clinical cure endpoint. Although this endpoint reflects real-world documentation, it resulted in high baseline response rates and a ceiling effect. Conversely, the strict three-domain sensitivity endpoint was highly dependent on complete follow-up laboratory and radiological documentation and generated sparse success counts. The complete-case analysis also failed to reproduce all primary findings. These discrepancies indicate that the estimated associations were sensitive to endpoint definition and missing-data handling.

Third, microbiological clearance was assessed only among patients with available follow-up cultures, introducing substantial selection bias. Because repeat cultures were not routinely obtained and may have been preferentially ordered for patients with persistent symptoms, more severe disease, suspected treatment failure, colonization, or resistant pathogens, microbiological clearance should not be interpreted as a definitive measure of comparative drug efficacy.

Finally, adverse events and mortality were rare. Safety analyses were sensitive to model choice, and mortality analyses were limited by small event counts and unmeasured goals-of-care factors. Therefore, all safety and mortality findings should be considered exploratory.

## 5 Conclusion

In this single-center real-world analysis of non-ICU hospitalized elderly patients with CAP, omadacycline showed a pragmatic clinical response comparable to tigecycline, higher than moxifloxacin, but lower than cefoperazone-sulbactam after IPTW adjustment and FDR correction. Omadacycline was associated with shorter hospital stay than tigecycline and cefoperazone-sulbactam, but longer hospital stay than moxifloxacin. Antibiotic duration was comparable to tigecycline, shorter than cefoperazone-sulbactam, but longer than moxifloxacin. Microbiological clearance was lower with omadacycline in the microbiologically evaluable subset, whereas recorded adverse events were generally fewer in the omadacycline group, although safety comparisons were limited by rare-event sparsity. No statistically significant difference in 30-day all-cause mortality was observed after weight trimming and GEE modeling.

Given the broad pragmatic endpoint, high baseline response rate, sensitivity to endpoint definition and missing-data handling, incomplete microbiological follow-up, rare safety and mortality events, and residual confounding inherent to retrospective data, these findings should be interpreted as exploratory associations rather than causal treatment effects. Future prospective multicenter studies using standardized CAP endpoints, frailty assessments, microbiological profiling, and long-term outcomes are required to validate individualized treatment strategies for elderly CAP patients.

## Data Availability

The original contributions presented in the study are included in the article/**Supplementary Material**. Further inquiries can be directed to the corresponding author.

## References

[B1] Arnold F. W. (2017). How antibiotics should be prescribed to hospitalized elderly patients with community-acquired pneumonia. Drugs Aging 34, 13–20. doi: 10.1007/s40266-016-0423-9 27928779

[B2] Berg J. K. Tzanis E. Garrity-Ryan L. Bai S. Chitra S. Manley A. . (2018). Pharmacokinetics and safety of omadacycline in subjects with impaired renal function. Antimicrob. Agents Chemother. 62, e02057-17. doi: 10.1128/AAC.02057-17 29158281 PMC5786750

[B3] Cillóniz C. Dominedò C. Pericàs J. M. Rodriguez-Hurtado D. Torres A. (2020). Community-acquired pneumonia in critically ill very old patients: a growing problem. Eur. Respir. Rev. 29, 190126. doi: 10.1183/16000617.0126-2019 32075858 PMC9488936

[B4] Corsonello A. Abbatecola A. M. Fusco S. Luciani F. Marino A. Catalano S. . (2015). The impact of drug interactions and polypharmacy on antimicrobial therapy in the elderly. Clin. Microbiol. Infect. 21, 20–26. doi: 10.1016/j.cmi.2014.09.011 25636922

[B5] Dequin P.-F. Torres A. Bassetti M. Ferrer M. Giamarellos-Bourboulis E. J. Martin-Loeches I. . (2026). Severe community-acquired pneumonia: current concepts and controversies. Intensive Care Med. 52, 118–130. doi: 10.1007/s00134-025-08252-x 41524797 PMC12852277

[B6] Elias C. Nunes M. C. Saadatian-Elahi M. (2024). Epidemiology of community-acquired pneumonia caused by S treptococcus pneumoniae in older adults: a narrative review. Curr. Opin. Infect. Dis. 37, 144–153. doi: 10.1097/QCO.0000000000001005 38323404

[B7] Ferreira-Coimbra J. Sarda C. Rello J. (2020). Burden of community-acquired pneumonia and unmet clinical needs. Adv. Ther. 37, 1302–1318. doi: 10.1007/s12325-020-01248-7 32072494 PMC7140754

[B8] Harhay M. O. Ratcliffe S. J. Halpern S. D. (2017). Measurement error due to patient flow in estimates of intensive care unit length of stay. Am. J. Epidemiol. 186, 1389–1395. doi: 10.1093/aje/kwx222 28605399 PMC5860585

[B9] Huang C.-T. Chen C.-H. Chen W.-C. Wang Y.-T. Lai C.-C. Fu P.-K. . (2022). Clinical effectiveness of cefoperazone-sulbactam vs. piperacillin-tazobactam for the treatment of pneumonia in elderly patients. Int. J. Antimicrob. Agents 59, 106491. doi: 10.1016/j.ijantimicag.2021.106491 34871744

[B12] Li S. Li L. Wang S. Wu H. (2025). Clinical characteristics and risk factors of hospital mortality in elderly patients with community-acquired pneumonia. Front. Med. (Lausanne) 12, 1512288. doi: 10.3389/fmed.2025.1512288 40177286 PMC11961441

[B13] Lodise T. Rodriguez M. Chitra S. Wright K. Patel N. (2021). Potential cost savings associated with targeted substitution of current guideline-concordant inpatient agents with omadacycline for the treatment of adult hospitalized patients with community-acquired bacterial pneumonia at high risk for Clostridioides difficile infections: results of healthcare-decision analytic model from the United States hospital perspective. Antibiotics (Basel) 10, 1195. doi: 10.3390/antibiotics10101195 34680776 PMC8532985

[B14] Ma S. Yang T. Gong H. Wang J. Chen K. Huang W. . (2026). Understanding antibiotic prescribing in the inpatient setting: a synthesis of evidence on determinants and interventions. Antimicrob. Resist. Infect. Control. 15, 56. doi: 10.1186/s13756-026-01726-7 41794790 PMC13081581

[B15] Mancinetti F. Marinelli A. Boccardi V. Mecocci P. (2024). Challenges of infectious diseases in older adults: from immunosenescence and inflammaging through antibiotic resistance to management strategies. Mech. Ageing Dev. 222, 111998. doi: 10.1016/j.mad.2024.111998 39447983

[B16] Metlay J. P. Waterer G. W. Long A. C. Anzueto A. Brozek J. Crothers K. . (2019). Diagnosis and treatment of adults with community-acquired pneumonia. An official clinical practice guideline of the American Thoracic Society and Infectious Diseases Society of America. Am. J. Respir. Crit. Care Med. 200, e45–e67. doi: 10.1164/rccm.201908-1581ST 31573350 PMC6812437

[B17] Putot A. Garin N. Rello J. Prendki V. European Study Group of Infections in the Elderly Patients (ESGIE) (2025). Comprehensive management of pneumonia in older patients. Eur. J. Intern. Med. 135, 14–24. doi: 10.1016/j.ejim.2025.02.025 40021428

[B19] Rodvold K. A. Pai M. P. (2019). Pharmacokinetics and pharmacodynamics of oral and intravenous omadacycline. Clin. Infect. Dis. 69, S16–S22. doi: 10.1093/cid/ciz309 31367744 PMC6669312

[B10] Sahuquillo-Arce J. M. Menéndez R. Méndez R. Amara-Elori I. Zalacain R. Capelastegui A. . (2016). Age-related risk factors for bacterial aetiology in community-acquired pneumonia. Respirology (Carlton Vic.) 21, 1472–1479. doi: 10.1111/resp.12851 27417291

[B20] Shi T. Denouel A. Tietjen A. K. Lee J. W. Falsey A. R. Demont C. . (2020). Global and regional burden of hospital admissions for pneumonia in older adults: a systematic review and meta-analysis. J. Infect. Dis. 222, S570–S576. doi: 10.1093/infdis/jiz053 30849172

[B21] Soraci L. Cherubini A. Paoletti L. Filippelli G. Luciani F. Laganà P. . (2023). Safety and tolerability of antimicrobial agents in the older patient. Drugs Aging 40, 499–526. doi: 10.1007/s40266-023-01019-3 36976501 PMC10043546

[B22] Stets R. Popescu M. Gonong J. R. Mitha I. Nseir W. Madej A. . (2019). Omadacycline for community-acquired bacterial pneumonia. N. Engl. J. Med. 380, 517–527. doi: 10.1056/NEJMoa1800201 30726692

[B23] Sun H. Hou X. Zhu Q. Liu J. Zhai Q. Shi K. (2025). Omadacycline pharmacokinetics: characteristics, contributing factors, and clinical significance. Drugs R. D. 25, 289–307. doi: 10.1007/s40268-025-00531-8 41379380 PMC12756217

[B24] Womack J. Kropa J. (2022). Community-acquired pneumonia in adults: rapid evidence review. Am. Fam. Physician 105, 625–630. 35704808

[B25] Xia J. Zhang D. Xu Y. Gong M. Zhou Y. Fang X. (2014). A retrospective analysis of carbapenem-resistant Acinetobacter baumannii-mediated nosocomial pneumonia and the *in vitro* therapeutic benefit of cefoperazone/sulbactam. Int. J. Infect. Dis. 23, 90–93. doi: 10.1016/j.ijid.2014.01.017 24726664

[B26] Yu M. Wang X. Wu S. Zhou Z. Liu Y. Chai Y. (2025). Comparison of efficacy and safety profiles between omadacycline and moxifloxacin in elderly patients with community-acquired pneumonia: a randomized, controlled trial. Clin. Pharmacol. Drug Dev. 15, e1627. doi: 10.1002/cpdd.1627 41267642

