# Supplementary material for: Efficacy and safety of Omadacycline in community-acquired pneumonia among elderly patients: a real-world evidence study
Source: Front Cell Infect Microbiol. 2026 Jul 31;16:1875598. doi: 10.3389/fcimb.2026.1875598 (PMC13472830; doi:10.3389/fcimb.2026.1875598)
Supplement: Supplementary file 1 [file Table1.docx]

Supplementary Material

**Supplementary Table 1.** Original sample size by age group and IPTW effective sample size

| Age Subgroup | Omadacycline | | Tigecycline | | Moxifloxacin | | Cefoperazone-sulbactam | | Overall | |
| --- | --- | --- | --- | --- | --- | --- | --- | --- | --- | --- |
|  | N | ESS | N | ESS | N | ESS | N | ESS | N | ESS |
| Age<75 years | 94 | 81.7 | 71 | 50 | 466 | 443.3 | 137 | 95.2 | 768 | 670.2 |
| Age≥75 years | 83 | 46.9 | 53 | 39.9 | 200 | 184.4 | 104 | 75 | 440 | 346.2 |

Note: IPTW, inverse probability of treatment weighting; N, unweighted raw sample size; ESS, effective sample size. The ESS represents the design-effect-adjusted population headcount following the allocation of stabilized inverse probability weights. It reflects the true statistical power and sample structural integrity remaining for subsequent robust inference testing within each specific demographic or clinical severity stratum.

**Supplementary Table 2.** Original sample size for the CURB-65 stratification and effective sample size for IPTW

| CURB-65 Subgroup | Omadacycline | | Tigecycline | | Moxifloxacin | | Cefoperazone-sulbactam | | Overall | |
| --- | --- | --- | --- | --- | --- | --- | --- | --- | --- | --- |
|  | N | ESS | N | ESS | N | ESS | N | ESS | N | ESS |
| CURB-65<2 | 106 | 87.5 | 35 | 32.3 | 345 | 336.9 | 57 | 54.4 | 543 | 511.1 |
| CURB-65≥2 | 71 | 52.3 | 89 | 79.1 | 321 | 297.7 | 184 | 168.5 | 665 | 597.6 |

Note: IPTW, inverse probability of treatment weighting; N, unweighted raw sample size; ESS, effective sample size. The ESS represents the design-effect-adjusted population headcount following the allocation of stabilized inverse probability weights. It reflects the true statistical power and sample structural integrity remaining for subsequent robust inference testing within each specific demographic or clinical severity stratum.

**Supplementary Table 3.** E‑value analysis for unmeasured confounding in the primary outcome comparisons

| Comparison | OR (95% CI) | E‑value (point estimate) | E‑value (lower CI bound) |
| --- | --- | --- | --- |
| Omadacycline vs Moxifloxacin | 0.522 (0.261–1.043) | 3.24 | 1 |
| Omadacycline vs Cefoperazone-sulbactam | 3.31 (1.02–10.78) | 6.08 | 1.16 |

OR, odds ratio; CI, confidence interval. Odds ratios were derived from IPTW-weighted logistic regression models. Point-estimate E-values and lower-bound E-values were calculated to quantify the potential sensitivity of the observed associations to unmeasured confounding. For OR values less than 1, the reciprocal OR was used for E-value calculation. The E-value for the confidence-limit closest to the null was calculated to evaluate the robustness of the confidence interval. An E-value close to 1 indicates that relatively weak unmeasured confounding could explain away the observed association. Therefore, although the point-estimate E-values were 3.24 and 6.08, the lower-bound E-values of 1.00 and 1.16 indicate that these observational associations remain vulnerable to unmeasured confounding and should be interpreted cautiously alongside the primary risk-difference analyses and sensitivity analyses.

**Supplementary Table 4.** Fisher’s test in safety analysis

| Comparison | treat_ae | control_ae | OR | OR_CI | P |
| --- | --- | --- | --- | --- | --- |
| Omadacycline vs Tigecycline | 1/177 | 8/124 | 0.08 | 0.08(0.00 to 0.63) | 0.004 |
| Omadacycline vs Moxifloxacin | 1/177 | 7/666 | 0.54 | 0.54(0.01 to 4.21) | >0.999 |
| Omadacycline vs Cefoperazone-sulbactam | 1/177 | 9/241 | 0.15 | 0.15(0.00 to 1.08) | 0.049 |

Note: OR, odds ratio; CI, confidence interval; ae, adverse events. Due to the extreme sparsity and low overall incidence of adverse events across the treatment cohorts, standard weighted regression models are prone to severe variance inflation. Consequently, this specific sensitivity analysis utilized an unweighted, exact non-parametric method (Fisher’s exact test) to provide a highly conservative baseline comparative safety metric free from probability-weighting artifacts.

**Supplementary Table 5.** The Firth Analysis in Safety Analysis

| comparison | OR | OR_CI | p_value |
| --- | --- | --- | --- |
| Omadacycline vs Tigecycline | 0.12 | 0.12(0.01 to 0.53) | 0.004 |
| Omadacycline vs Moxifloxacin | 0.75 | 0.75(0.08 to 3.45) | 0.738 |
| Omadacycline vs Cefoperazone-sulbactam | 0.21 | 0.21(0.02 to 0.91) | 0.036 |

Note: OR, odds ratio; CI, confidence interval. To further address the rare-event data sparsity (e.g., single-digit adverse event counts) that typically induces data separation and destabilizes standard maximum likelihood estimation in logistic regression, a Firth's penalized-likelihood logistic regression was executed. This advanced methodological approach systematically applies a penalization term to mitigate small-sample bias and mathematical separation, thereby yielding robust, finite safety odds ratios that validate the primary weighted risk differences.
